# OSBP-Related Proteins (ORPs) in Human Adipose Depots and Cultured Adipocytes: Evidence for Impacts on the Adipocyte Phenotype

**DOI:** 10.1371/journal.pone.0045352

**Published:** 2012-09-21

**Authors:** You Zhou, Marius R. Robciuc, Martin Wabitsch, Anne Juuti, Marja Leivonen, Christian Ehnholm, Hannele Yki-Järvinen, Vesa M. Olkkonen

**Affiliations:** 1 Minerva Foundation Institute for Medical Research, Helsinki, Finland; 2 National Institute for Health and Welfare, Public Health Genomics Unit, Helsinki, Finland; 3 Division of Paediatric Endocrinology and Diabetes, Department of Paediatrics and Adolescent Medicine, University of Ulm, Ulm, Germany; 4 Department of Surgery, Helsinki University Central Hospital, Helsinki, Finland; 5 Department of Medicine, University of Helsinki, Helsinki, Finland; 6 Institute of Biomedicine, Anatomy, University of Helsinki, Helsinki, Finland; Simon Fraser University, Canada

## Abstract

Oxysterol-binding protein (OSBP) homologues, ORPs, are implicated in lipid homeostatic control, vesicle transport, and cell signaling. We analyzed here the quantity of ORP mRNAs in human subcutaneous (s.c.) and visceral adipose depots, as well as in the Simpson-Golabi-Behmel syndrome (SGBS) adipocyte cell model. All of the ORP mRNAs were present in the s.c and visceral adipose tissues, and the two depots shared an almost identical ORP mRNA expression pattern. SGBS adipocytes displayed a similar pattern, suggesting that the adipose tissue ORP expression pattern mainly derives from adipocytes. During SGBS cell adipogenic differentiation, ORP2, ORP3, ORP4, ORP7, and ORP8 mRNAs were down-regulated, while ORP11 was induced. To assess the impacts of ORPs on adipocyte differentiation, ORP3 and ORP8, proteins down-regulated during adipogenesis, were overexpressed in differentiating SGBS adipocytes, while ORP11, a protein induced during adipogenesis, was silenced. ORP8 overexpression resulted in reduced expression of the aP2 mRNA, while down-regulation of adiponectin and aP2 was observed in ORP11 silenced cells. Furthermore, ORP8 overexpression or silencing of ORP11 markedly decreased cellular triglyceride storage. These data identify the patterns of ORP expression in human adipose depots and SGBS adipocytes, and provide the first evidence for a functional impact of ORPs on the adipocyte phenotype.

## Introduction

Bioactive lipid signaling molecules are key players in cell regulation, and disturbances in signaling lipids such as oxysterols, sphingolipids/ceramides, fatty acid derivatives and diacylglycerols, are associated with metabolic and cardiovascular diseases [1]–[3].

Oxysterols are 27-carbon oxygenated products of cholesterol that arise through enzymatic or non-enzymatic oxidation processes, or are absorbed from the diet [4], [5]. As ligands of sterol-regulated transcription factors and intermediates in the biosynthesis of bile acids and steroid hormones, oxysterols control gene expression in lipid metabolism, regulate immune and inflammatory responses, and modify cellular calcium signaling [4], [6], [7]. Oxysterols are present at low concentrations in tissues and their circulating concentrations are altered in obesity, metabolic syndrome and cardiovascular disease [8]–[12]. However, there is very limited information available on the role of oxysterols or the proteins mediating their biological effects in adipose tissue [13]. Wamil et al. [14] reported that the oxysterol 7-ketocholesterol inhibits the activity of glucocorticoids and impairs the differentiation of mouse 3T3-L1 adipocytes, while Kha et al. [15] failed to observe this effect in human mesenchymal stem cells. 22(R)-, 20(S)- and 20(S)-hydroxycholesterol (OHC) were found to inhibit adipocytic and to promote osteogenic differentiation of the stem cells through the Hedgehog signaling pathways [15]–[18]. Furthermore, Baranova et al. [19] found that CH25H, a cholesterol hydroxylase catalysing oxysterol synthesis, was significantly down-regulated in the visceral adipose tissue of obese individuals in comparison to non-obese ones, suggesting a distinct functional role of 25-OHC in adipose tissue.

Oxysterol-binding protein (OSBP) and its homologues designated OSBP-related proteins (ORPs) are lipid binding proteins, the β-barrel-like ligand-binding domain of which can accommodate lipids such as oxysterols, cholesterol, or phosphatidylinositol-4-phosphate [20]–[22]. They are proposed to regulate cellular lipid homeostasis and to act as sterol transporters and signaling sensors. OSBP, the founder member of the protein family, shows high affinity for a variety of oxysterols [23]. As a sterol sensor, OSBP regulates ceramide transport from the endoplasmic reticulum (ER) to the Golgi apparatus, mediated by the ceramide transporter, CERT [24], [25]. Interestingly, OSBP also acts as a cholesterol-dependent scaffold for protein phosphatases in the extracellular signal-regulated kinase (ERK) signaling pathway [26]. Moreover, OSBP has the capacity to regulate the insulin induction of SREBP-1c and hepatic lipogenesis [27]. The ORP protein/gene family in humans and mice consists of a large number of proteins encoded by 12 genes, which serve diverse functions in cellular lipid metabolism and signaling [28]–[30]. Several members of the ORP family have been putatively connected with metabolic diseases: ORP8 was suggested to regulate insulin signaling in mouse models of obesity [31] and lipid levels in mouse plasma and liver tissue [32]. Interestingly, ORP11 was found to be abundantly expressed in visceral adipose tissue and was associated with cardiovascular risk factors in obese subjects with metabolic syndrome [33]. Furthermore, it was reported that the expression levels of OSBPL11 were significantly different in adipose tissue between the high and low responders to caloric restriction [34]. However, there are no data comparing the expression of OSBP/ORP in human visceral and subcutaneous adipose tissues, or assessing the functional role of ORPs in adipocytes. In the present study we analyze the expression patterns of ORP mRNAs in human subcutaneous and visceral adipose depots, as well as in Simpson-Golabi-Behmel syndrome (SGBS) cells. These cells represent a human preadipocyte model that is neither transformed nor immortalized, shows a high capacity for adipose differentiation, and is used as a model for normal adipocyte differentiation [35], [36]. Moreover, we examine the functional impacts of selected ORPs (ORP3, ORP8 and ORP11) on SGBS adipocyte differentiation and triglyceride storage.

## Methods

### Antibodies and Other Reagents

The rabbit ORP2, ORP3, ORP8 and ORP11 antibodies have been described previously [37]–[40]. Rabbit OSBP antibody was a gift from Dr. Maria Antonietta De Matteis (Telethon Institute of Genetics and Medicine, Naples, Italy). Anti-ORP9 rabbit antibody was kindly provided by Prof. N. Ridgway (Dalhousie University, Halifax, Nova Scotia, Canada). Anti-β-actin and anti-aP2 rabbit polyclonal antibodies were purchased from Sigma-Aldrich (St. Louis, MO).

**Table 1:** Oligonucleotide primers for mRNA quantification by quantitative real-time reverse transcription-PCR.

mRNA	Forward primer 5′-3′	Reverse primer 5′-3′
OSBP	GATCCATCAGGAAAAGTCCAC	CAGTGCCACTTTCCCAAGCA
ORP1S	GGTCCTCGGATCTGGCCCA	ACTCAGGGACCTTTCGGACTC
ORP1L	TGATTGCCTTAATCTCTTCACC	ACTCAGGGACCTTTCGGACTC
ORP2	ACCACCTGAGAAAGGCCAAGC	CTCCAGCTCGTTGAGGCTCAC
ORP3	GCATCTAGCTACTACCGAGCT	CACATGGGTTGTGCCAATTGGA
ORP4	AAGCTCTGGATCGACCAGTCA	CTGGGACTGCTATGCATGACC
ORP5	GTGCCGCTGGAGGAGCAGAC	AGGGGCTGTGGTCCTCGTATC
ORP6	GGATACTGCTCCACCTATTTCA	ACAGGCAGGATTTCCATCGAC
ORP7	CGGCCTACTCCTCCACATACC	ACTGTTCCCACAGGCACAATC
ORP8	GAAGAACAGGGAGATTTTGAATCA	TCCTGTGAGTGGATCAAGTTC
ORP9	CATCTTCCACACTAAACCCTTC	CTCGTTCTGATCTTCCAACTTC
ORP10	CTCAGCGACAGTGATATTCCAC	AGGTCTGATCTTCTTGGGATAC
ORP11	CACATTTTCTCTACCCTGTGCA	CCCTTGCACTCTGCATACCAC
Adiponectin	GGCCGTGATGGCAGAGAT	CCTTCAGCCCGGGTAC
aP2	GCTTTTGTAGGTACCTGGAAACTT	ACACTGATGATCATGTTAGGTTTGG
Leptin	GCCCTATCTTTTCTATGTCC	TCTGTGGAGTAGCCTGAAG
PPARγ	GATCCAGTGGTTGCAGATTACAA	GAGGGAGTTGGAAGGCTCTTC
SDHA	CATGCTGCCGTGTTCCGTGTGGG	GGACAGGGTGTGCTTCCTCCAGTGCTCC
β-actin	GACAGGATGCAGAAGGAGATT	TGATCCACATCTGCTGGAAGG

### Western Blot Analysis

Protein samples for SDS-PAGE were prepared by homogenizing cultured cells in 250 mM Tris-HCl, pH 6.8, 8% SDS, protease inhibitor cocktail (Roche Diagnostics, Mannheim, Germany). The crude extracts were cleared by centrifugation in a microfuge at 13,000 rpm for 3 min, and the protein concentration of the supernatants was determined by the DC assay (BioRad, Hercules, CA). The proteins were electrophoresed on Laemmli gels and electrotransferred onto Hybond-C Extra nitrocellulose membranes (Amersham Biosciences, Piscataway, NJ) for different periods of time according to the protein size. Unspecific binding of antibodies was blocked with, and all antibody incubations were carried out in 5% fat free powdered milk in 10 mM Tris-HCl, pH 7.4, 150 mM NaCl, and 0.05% Tween 20. The bound primary antibodies were visualized with horseradish peroxidase-conjugated goat anti-rabbit IgG (Bio-Rad) and the enhanced chemiluminescence system (Thermo Scientific, Rockford, IL). The expression levels were quantified by densitometry using ImageJ (http://rsbweb.nih.gov/ij/), and the data was normalized according to the β-actin signal.

**Figure 1:**
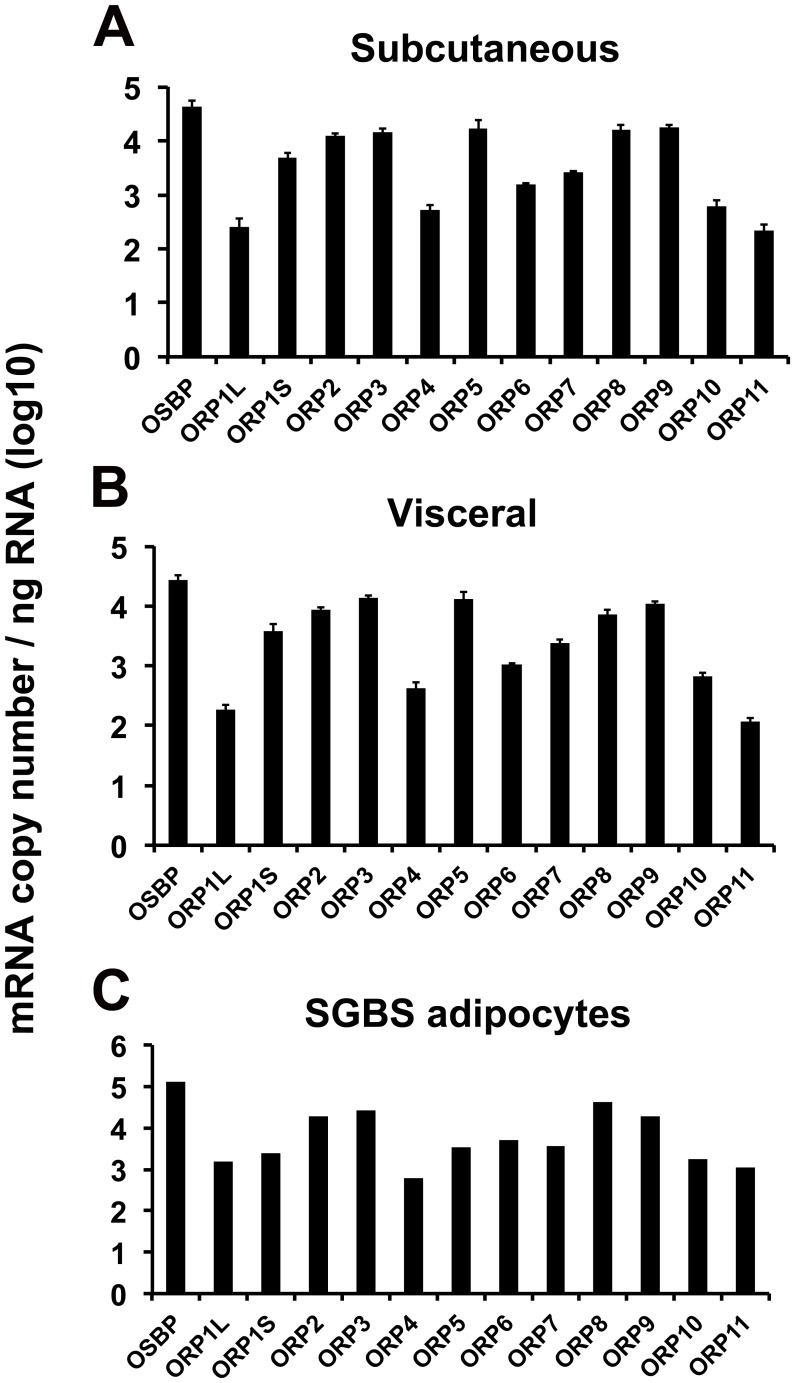
Copy numbers of OSBP/ORP mRNAs in human adipose tissues and SGBS adipocytes. The mRNAs were quantified by qPCR using the corresponding cDNAs as calibrators (see Materials and Methods), and the mRNA quantities are presented on a log10 scale as copies/ng total RNA. A. Subcutaneous adipose tissue; B. Visceral adipose tissue. The data represents mean ± S.E., n = 4. C. Simpson-Golabi-Behmel syndrome (SGBS) adipocytes after 22-day differentiation. The mean from a single experiment carried out in triplicate is shown.

**Figure 2:**
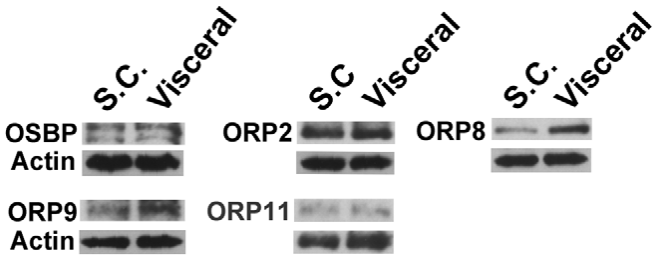
Detection of ORP proteins in human subcutaneous (S.C.) and visceral adipose tissue specimens. Protein extracts of human adipose tissues (20****µg/lane) were resolved on SDS-PAGE and analyzed by Western blotting with antibodies against OSBP, ORP2, ORP8, ORP9, ORP11, and β-actin, as indicated.

### Cell Culture and Lentiviral/adenoviral Transduction

Human Simpson-Golabi-Behmel syndrome (SGBS) pre-adipocytes [35], [36] were cultured in Dulbecco’s modified Eagle’s medium (DMEM)/Ham’s F12 medium (Invitrogen, Paisley, UK) containing 10% fetal bovine serum (FBS; Sigma-Aldrich), 66 nM biotin (Sigma-Aldrich), 33 nM D-pantothenic acid (Sigma-Aldrich) and 2% penicillin/streptomycin. Cells were seeded onto 12-well plates and grown to 80%–90% confluency for differentiation. To induce differentiaton, the cells were washed three times with PBS and changed to serum-free DMEM/F12 medium supplemented with 0.01 mg/ml human apo-transferrin (Sigma-Aldrich), 20 nM insulin, 100 nM cortisol (Sigma-Aldrich), 0.2 nM triiodothyronine (Sigma-Aldrich), 25 nM dexamethasone (Sigma-Aldrich), 250 µM 1-methyl-3-isobutyl-xanthine (IBMX, (Sigma-Aldrich) and 2 µM rosiglitazone (Sigma-Aldrich) for 4 days. The cells were subsequently differentiated in serum-free DMEM/F12 medium containing 20 nM insulin, 100 nM cortisol, and 0.2 nM triiodothyronine for additional 18 days. The differentiation was monitored by quantifying Oil Red O stainable lipid droplets and by measuring by qPCR the mRNA levels of PPARγ, aP2, leptin, and adiponectin.

**Figure 3:**
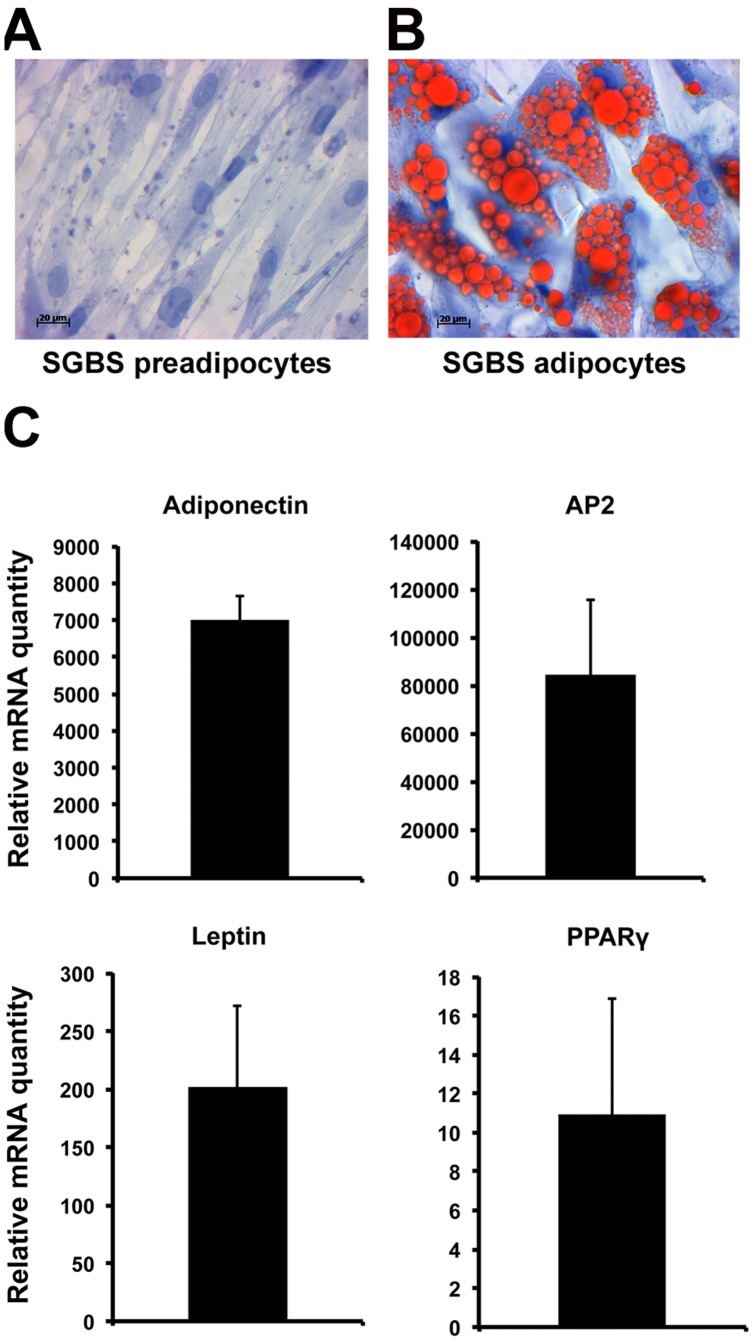
Adipogenic differentiation of SGBS cells. Haematoxylin and Oil Red O staining of SGBS preadipocytes (A) and adipocytes differentiated for 22 days (B). C. Relative mRNA levels of the adipocyte differentiation markers adiponectin, aP2, leptin and PPARγ in SGBS adipocytes as compared to preadipocytes. The bars indicate fold-change, mean ± S.E. (n = 3).

pGIPZ-ORP11 shRNA construct (Cat. no. RHS4430-98704927) and pGIPZ empty vector were purchased from OpenBioSystems/Thermo Scientific (Huntsville, AL). Lentiviral particles were generated by transfection of the shRNA and packing plasmids pHelper and pEnvelope to HEK 293FT cells using the FuGENE HD Reagent (Roche Diagnostics). After three days’ transfection, the viral supernantant was filtered through a 0.45 µm PVDF filter and further concentrated by ultracentrifugation for 2 hrs in an SW28 rotor at 19,400 rpm, 4°C. ORP3 and ORP8 adenoviruses were produced as described [32], [41]. SGBS preadipocytes were transduced with the shRNA lentiviruses in the presence of 8 µg/ml hexadimethrine bromide, followed by 0.5 µg/ml puromycine antibiotic selection of stable cell pools. For adenoviral transduction, SGBS adipocytes were transiently transduced for 72 hrs at a m.o.i. of 50 I.U./cell, on day 10 during the differentiation. The silencing or overexpression of target ORPs was verified by qPCR and Western blotting.

**Figure 4:**
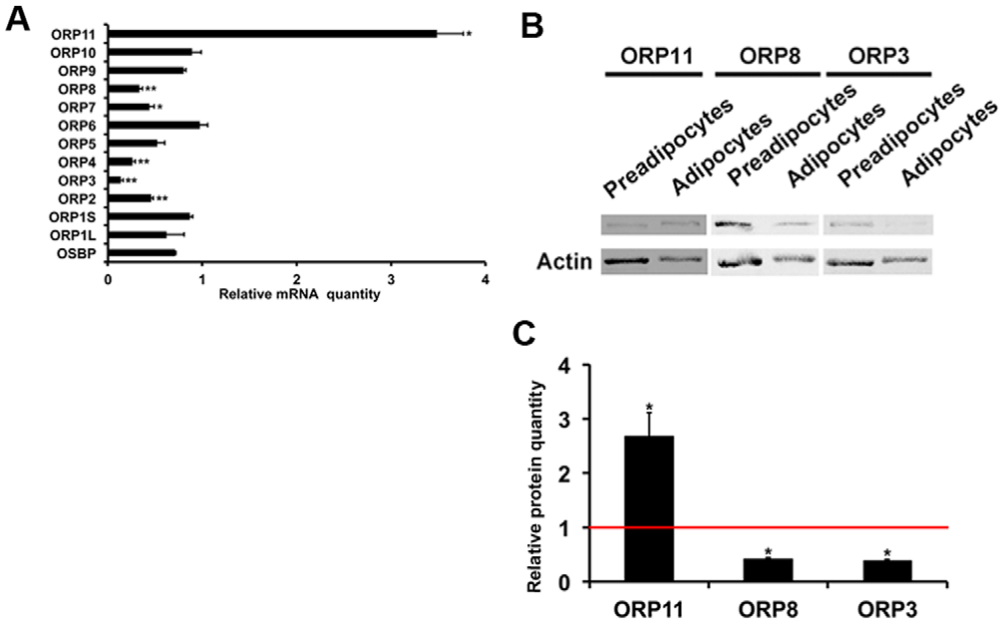
Changes in ORP mRNA and protein expression upon SGBS cell adipogenic differentiation. A. Quantities of the indicated ORP mRNAs on day 22 of differentiation. The results are presented as fold change relative to preadipocytes. The data represents mean ± S.E., n = 3−4, *p<0.05, **p<0.01. B. Western blot analysis of the ORP11, ORP8, and ORP3 proteins in SGBS preadipocytes and adipocytes (day 22). C. Quantification of the Western data by densitometric analysis. The ORP11, ORP8 and ORP3 signals were normalized for that of β-actin. The data represents mean ± S.E., n = 3−4, *p<0.05.

**Figure 5:**
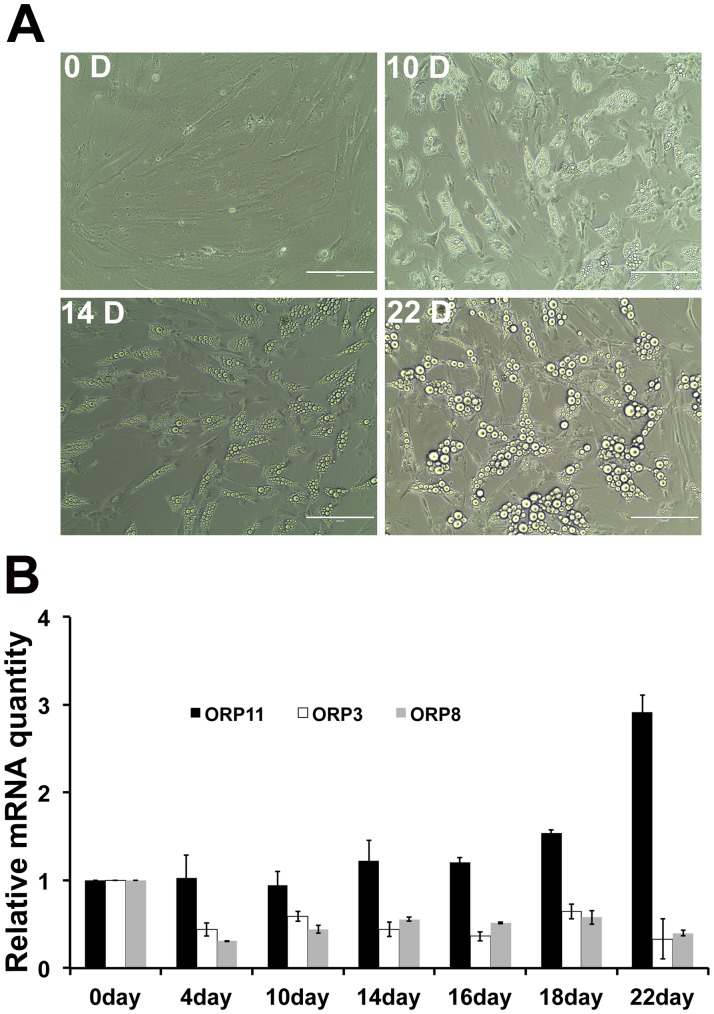
Time course of ORP11, ORP3, and ORP8 mRNA changes upon SGBS cell adipogenic differentiation. A. Phase contrast images of SGBS preadipocytes (0 D) and differentiating adipocytes on days 10, 14, and 22. Bars, 200 µm. B. The ORP mRNA levels were quantified at the differentiation time points indicated, as fold changes relative to preadipocytes (day 0). The data represents mean ± S.E., n = 3.

### Bioinformatics Analysis of ORP mRNA Expression

The microarray dataset of Human U133A/GNF1H Gene Atlas from BioGPS web site (http://biogps.org/downloads/) was used for the analysis. We focused on the OSBP/ORP probe sets in different tissues; their information was extracted separately. Some probe sets judged to be non-functional were eliminated if their signals were throughout the tissue selection at or close to the median value across all the samples for the specific probe set. The functionality of probe sets was further critically evaluated based on a comparison to the published results on ORP mRNA or protein expression.

**Figure 6:**
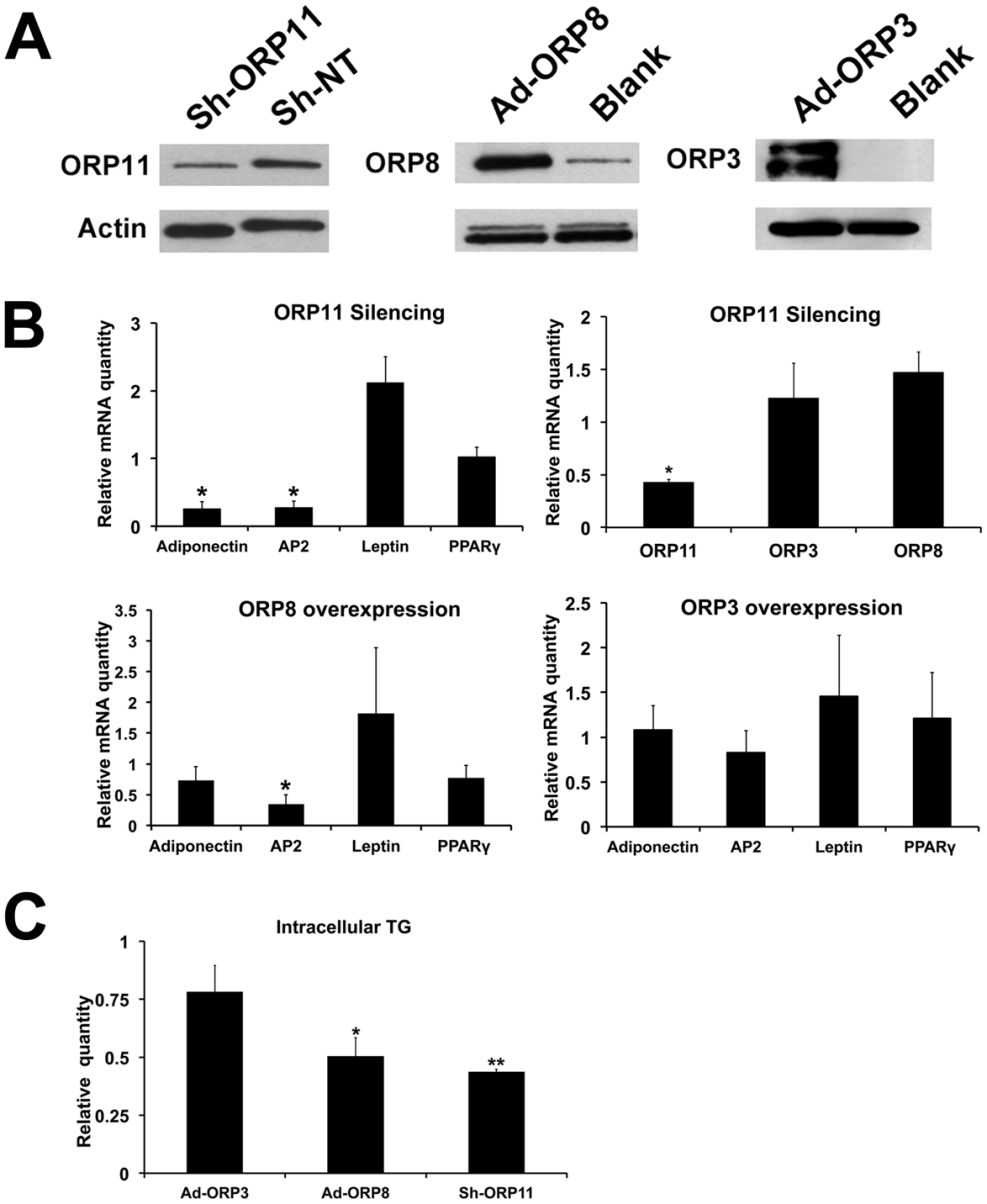
Impacts of ORP manipulation on SGBS cell adipogenic differentiation. ORP8 or ORP3 were overexpressed by infecting cells on day 10 with control (Blank) or ORP (Ad-ORP8, Ad-ORP3) adenoviral vectors, and collected for analysis on day 13. SGBS preadipocytes were transduced with an ORP11 shRNA (Sh-ORP11) or non-targeting (Sh-NT) shRNA lentivirus, followed by differentiation for 22 days. A. Western blots of total cell protein (10 µg/lane); ORP11, after 22 days of differentiation; ORP8 and ORP3, after 72 h of adenoviral transduction. The blots were probed with anti-β-actin as a loading control. B. The impacts of ORP11 silencing or ORP8/ORP3 overexpression on the mRNA levels of adipocyte differentiation markers adiponectin, aP2, leptin and PPARγ. In cells with ORP11 stably silenced also ORP8 and ORP3 mRNAs were quantified. The results are shown as fold changes relative to cells infected with the corresponding control viruses, and represent mean ± S.E., n = 3; *p<0.05. C. The cellular triglyceride concentration was measured by using an enzymatic assay. The results were normalized for total cell protein and are presented relative to cells infected with the corresponding control viruses (mean ± S.E., n = 3; *p<0.05, **p<0.01).

### Human Adipose Tissue Specimens

Human subcutaneous and visceral adipose tissue specimens were obtained from four morbidly obese patients upon bariatric surgery at Peijas Hospital, Finland. The patients were female and their ages ranged from 33 to 64 yrs. Their average BMI was averaged 46.9 kg/m^2^ (range 43.7 to 50.8 kg/m^2^) and waist to hip ratio 0.91 (range 0.88–0.96).

The subjects were volunteers recruited according to a permission (28/13/03/02/2010) from the Medicinal Ethics Committee of the Helsinki and Uusimaa Hospital Distric. The Finnish Ethics Committees obey the current Finnish law. The Medical Research Act No. 488/1999 takes into consideration statutory regulations and directives (Mostly Directives 2001/20/EC and 2005/28/EC) within EC area. The Ethics committee operates according to the principles of good clinical research practice (ICH-GCP-E6) and in accordance with the international obligations concerning the status of re- search subjects and the rules and guidelines that govern research (Medical Research Act 488/1999, chapter 2 a (23.4.2004/295), section 5 and 10a). The Medicinal Ethics Committee of the Helsinki and Uusimaa Hospital District is registered with the HHS and follows ICH-GCP-E6 in reviewing all research in the Helsinki and Uusimaa Hospital District involving human subjects. Minerva Foundation Institute for Medical Research does not have its own ethics committee. All clinical research carried out by Minerva researchers occurs at the clinics of the Helsinki and Uusimaa Hospital District, and ethical approvals for these studies have to be exclusively obtained from the Medicinal Ethics Committee of this Hospital District. No research was conducted outside our country of residence. The patients signed a written consent after receiving detailed information on the study protocol and after having an adequate time for deliberation. All procedures and possible hazards, risks and discomforts were explained to the subjects both orally and in writing. The signed forms are archived in our study documentation.

### Quantitative RT-PCR (qPCR) Analyses

Total RNA (RNeasy Lipid Tissue Mini kit, QIAGEN, Germantown, MD) was extracted from s.c. and visceral adipose tissues of 4 patients. The RNAs were reverse transcribed by using the VILO kit (Invitrogen, Carlsbad, CA) and subjected, by using primer sets specific for 13 major ORP transcripts (Table 1), to qPCR analysis using SYBR Green I Master Mix (Roche Diagnostics) and a Roche LightCycler 480 II instrument.

Human ORP open reading frames were cloned into pcDNA4HisMaxC (Invitrogen, Carlsbad, CA) as described before [38]–[40], [42], [43]. pEGFP-OSBP was a gift from Dr. Maria Antonietta De Matteis (Telethon Institute of Genetics and Medicine, Naples, Italy). The full-length ORP cDNA inserts were excised from the constructs and used as calibrators to quantify the corresponding mRNA copy numbers in human adipose specimens and SGBS adipocytes. A copy number standard curve was created from a dilution series of each ORP cDNA and used as a reference template in the same PCR runs. C_T_ values given by the tissue and SGBS cells were further converted to copy numbers using the standard curve [42]. Relative mRNA quantification of ORPs, adiponectin, aP2, leptin, and PPARγ was carried out by using the ΔΔC_T_ method with succinate dehydrogenase subunit alpha (SDHA) and β-actin as reference housekeeping mRNAs (Table 1).

### Oil Red O Staining of Neutral Lipids

Cells were washed with PBS, fixed by using 10% formalin for 30 min and then gently rinsed with water. After incubation with 60% isopropanol for 5 min, the cellular neutral lipids were stained by using 1.8 mg/ml Oil Red O in 60% isopropanol. Cells were rinsed with water and stained by using Hematoxylin at room temperature for 1 min.

### Assay for Cellular Triglyceride Content

After lentiviral or adenoviral transduction, SGBS adipocytes were lysed in RIPA buffer containing 50 mM Tris-HCl (pH 7,4), 1% NP-40, 0.25% Na-deoxycholate, 150 mM NaCl, 1 mM EDTA, 1% SDS and protease inhibitor cocktails (Roche Diagnostics). The supernatant was harvested after centrifugation for 5 min, and 10 µl samples as well as the glycerol standards (2300, 1150, 575, 287.5, 143.75 and 71.88 µmol/L) were subsequently mixed with 200 µl of the GPO-PAP Triglyceride kit (Cobas, Roche/Hitachi) reagent on a 96-well plate. After incubation at room temperature for 20 min, colorimetric assay was performed at 510 nm by using a Victor multilabel analyzer (Wallac, Turku, Finland). The triglyceride concentrations were normalized for total cell protein determined with the DC assay (BioRad).

### Statistical Analysis

The data are presented as mean ± standard error of mean (SEM). Student’s T-test was applied for comparisons between groups of data points. All statistical tests were two-sided and p values <0.05 were considered significant.

## Results

### OSBP/ORP Copy Numbers in Human Adipose Depots and SGBS Adipocytes

OSBP/ORP mRNA copy numbers were determined in s.c. and visceral adipose tissue biopsy specimens from 4 obese female patients as well as in SGBS adipocytes. The OSBP/ORP expression patterns of the s.c. and visceral fat depots were almost identical (Fig. 1A, B), and the SGBS adipocytes displayed a highly similar pattern (Fig. 1C), consistent with the view that the mRNA signals in human adipose tissue specimens mainly derive from adipocytes, while the adipose tissues also contain significant numbers of other cell types such as fibroblasts, macrophages, and endothelial cells [44], the ORP mRNAs of which could disturb interpretation of the expression data. Of the ORPs the mRNAs of which were abundant in the adipose depots, we were able to detect by Western blotting of total protein specimens OSBP, ORP2, ORP8, and ORP9, at relatively equal levels in the two depots (Fig. 2). Even though the ORP11 mRNA was present at relatively low levels in the adipose tissues, the protein was detectable on Western blots due to the high sensitivity of our antiserum.

By applying Human U133A/GNF1H Gene Atlas dataset, we investigated OSBP/ORPs expression profiles across the human tissues with the help of a heat-map (Fig. S1). This data set suggests, in accordance with earlier reports [45], [46], that many ORP mRNAs, such as OSBP, ORP4, and ORP9, are expressed at relatively even levels in a wide variety of tissues and cell types. The analysis also suggested that, even though the ORP11 mRNA is not abundant in adipose tissues on an absolute scale, it is present in adipocytes at markedly higher levels than in the other tissues/cell types, with the exception of CD14(+) monocytes, in which the message also is abundant.

### OSBP/ORP Expression during SGBS Adipocyte Differentiation

To assess the OSBP/ORP expression level during adipocyte differentiation, we employed the SGBS preadipocyte-adipocyte model. After 22 days of differentiation, approximately 80% of the cells were filled by large lipid droplets (Fig. 3A,B). As molecular markers for adipocytic differentiation, we monitored by qPCR the expression levels of adiponectin, aP2, leptin and PPARγ; These mRNAs increased dramatically during the 22-day incubation (Fig. 3C), similar to previous reports [35], [36], evidencing for successful adipogenic differentiation.

We analyzed ORP mRNA relative quantities in the preadipocytes and adipocytes on day 22. Quantitative RT-PCR analyses revealed that the ORP2 (−54%), ORP3 (−86%), ORP4 (−74%), ORP7 (−56%), and ORP8 (−67%) messages were significantly down-regulated during SGBS cells adipogenesis, while the ORP11 mRNA was elevated 3.5-fold (Fig. 4A). Of these we selected for further functional analysis ORP3, ORP8, and ORP11, which we have investigated in detail in previous studies [32], [39]–[41], and for which we have high quality molecular and immunological tools available. The observed changes in ORP3, ORP8 and ORP11 expression were confirmed at the protein level by Western blotting (Fig. 4B). Here, we observed upon SGBS adipocytic differentiation a 2-fold increase of ORP11 protein, a 55% reduction of ORP8, and a 60% reduction of ORP3 (Fig. 4C).

### Time Course of ORP3, ORP8 and ORP11 Expression Changes

To determine the time course of the alterations in the mRNA levels of ORP3, ORP8, and ORP11 during SGBS cell adipogenesis, we analyzed the mRNA expression levels on days 0, 4, 10, 14, 16, 18, and 22 of differentiation. The number and size of lipid droplets in the SGBS cells increased gradually (Fig. 5A). ORP3 and ORP8 were significantly down-regulated already between days 0 and 4, and no further decrease was detected at the later time points. In contrast, the ORP11 mRNA was found to be induced at late time points, the elevation occurring between days 16 and 22 (Fig. 5B).

### Effects of ORP Silencing/overexpression on Adipocyte Differentiation

To elucidate whether ORP3, ORP8, and ORP11 influence adipogenesis in SGBS cells, we silenced ORP11, the protein up-regulated upon SGBS adipocyte differentiation, by using a lentiviral shRNA expression vector. Correspondingly, we overexpressed two ORPs down-regulated upon adipocyte differentiation, ORP3 and ORP8, by using recombinant adenoviral vectors.

We first established SGBS preadipocytes transduced with a lentivirus expressing ORP11 shRNA; 60–70% reduction of the ORP11 protein was observed in SGBS adipocytes differentiated from the transduced preadipocytes, as compared to the controls transduced with a non-targeting shRNA lentivirus (Fig. 6A). When these cells were differentiated for 22 days, and the mRNAs for the adipogenesis markers were quantified, we observed a significant reduction of adiponectin and aP2 mRNAs in the ORP11 knock-down cells as compared to the controls (Fig. 6B). Reduction of ORP11 did not significantly alter the mRNA expression of ORP3 or ORP8.

ORP3 or ORP8 were overexpressed for 3 days starting on day 10 of SGBS adipocytic differentiation, a time at which the endogenous ORP3 and −8 mRNAs were firmly down-regulated and the cytoplasmic lipid droplets were rapidly growing (see Fig. 5). The transduction resulted in a robust increase of the cellular ORP3 or ORP8 proteins (Fig. 6A). ORP8 overexpression resulted in a significant down-regulation of the aP2 mRNA (by 61%), as compared to cells transduced with a control adenovirus. However, ORP3 overexpression did not significantly affect the adipogenesis marker mRNA levels (Fig. 6B).

To monitor the putative impacts of the ORP manipulations on the hallmark of the adipocyte phenotype, neutral lipid accumulation, we quantified triglyceride concentrations in SGBS cells in which ORP11 was silenced, on day 22 of differentiation, and in cells transduced for 3 days with ORP3 or ORP8 adenoviruses as specified above. This analysis revealed that silencing of ORP11 and overexpression of ORP8 markedly (by >50%) reduced the storage of cellular triglycerides as compared to cells transduced with the appropriate control viruses, while ORP3 overexpression did not significantly influence the cellular triglyceride accumulation (Fig. 6C).

## Discussion

Members of the ORPs family are thought to act as sterol sensors that relay information to the cellular machineries maintaining lipid homeostasis, energy balance and cell signaling. In the present study, we describe OSBP/ORP expression patterns in human adipose depots and SGBS preadipocytes/adipocytes. Furthermore, we present evidence for a functional impact of ORPs on the adipocyte phenotype.

OSBP/ORPs displayed a similar expression pattern in subcutaneous and visceral adipose tissues from morbidly obese patients. However, the expression profiles across different ORPs were markedly uneven, and resembled that in SGBS adipocytes. This suggests that the distinct ORP expression pattern observed in the human adipose tissues mainly reflects the adipocytic ORP profile, and is not strongly disturbed by other cell types present in the fat tissues, such as fibroblasts, macrophages, and endothelial cells [44]. Bioinformatic analysis suggested that, despite a relatively low mRNA copy number, ORP11 has an expression hotspot in adipocytes, unlike the other ORPs. In keeping with this, ORP11 mRNA and protein concentrations were up-regulated upon SGBS cell adipocytic differentiation while ORP2, ORP3, ORP4, ORP7 and ORP8 mRNAs (verified for ORP3 and ORP8 also at the protein level) were found to be down-regulated in the SGBS adipocytes as compared to preadipocytes.

ORP11 was reported to be up-regulated in the visceral adipose tissue of obese Canadian men with metabolic syndrome compared to men without the metabolic syndrome [33]. We have previously found ORP11 to localize at the Golgi-late endosome interface in HEK293 cells, and demonstrated its interaction with the related ORP9 [40], a protein implicated in sterol sensing or transport between the endoplasmic reticulum and the Golgi complex [47]. However, the precise function of ORP11 remains poorly understood. Knock-down of ORP11 in the SGBS cell model resulted in decreased expression of adiponectin and aP2. Adiponectin is an adipokine highly expressed in the white adipose tissue. Because of its protective role against chronic inflammation, insulin resistance, weight gain, obesity and cardiovascular disease, adiponectin is evaluated as an important potential target for therapy development [48]. aP2, also designated as FABP4, is a 15-kD cytosolic fatty acid binding protein present in adipocytes and macrophages. The protein plays a key role in glucose and lipid homeostasis, and its expression is regulated by fatty acids and insulin [49]–[51]. The expression level of aP2 was suggested to predict the risk of metabolic syndrome, type 2 diabetes and the development of atherogenic dyslipidemia [52]–[54]. In addition to impacts on adiponectin and aP2 expression, silencing of ORP11 resulted in an impairment of triglyceride storage in the SGBS adipocytes. During the SGBS differentiation time course, ORP11 was induced at late time points (16–22 days). Therefore, ORP11 most likely plays a role during late stages of adipogenic differentiation and possibly in the maintenance of the mature adipocyte phenotype.

ORP8 is abundantly expressed in CD14(+) monocytes (Fig. S1) and in tissue macrophage [39], bringing up the possibility that its expression in the adipose depots of the patients studied could in part derive from monocyte-macrophages abundant in the inflamed adipose tissue of obese subjects [55]. However, the abundant expression of ORP8 mRNA we detected in SGBS adipocytes and the data at Human U133A/GNF1H Gene Atlas suggest that the human adipose depot ORP8 mRNA derives to a large extent from adipocytes. Together with our recent data on ORP8 function [32], [56], [57], these observations suggest that the protein mediates lipid signals and fine-tunes the adipocyte lipid metabolism. Consistent with this notion, ORP8 overexpression in adipocytes significantly reduced the mRNA expression of aP2 and attenuated cellular triglyceride storage. Interestingly, Jordan et al. [31] reported that ORP8, as a target of miR-143, has a function in insulin signaling in mouse hepatocytes. These observations are consistent with the idea that ORP8 manipulation may, via modification of membrane microdomain organization, impact signal transduction events. We therefore envision that ORP8 overexpression might attenuate insulin stimulation of the adipogenic process. Further studies on the putative connection of ORP8 with the insulin signaling pathways in adipocytes are therefore warranted.

ORP3 overexpression did not significantly alter the adipocyte differentiation marker mRNA levels or the triglyceride storage in SGBS adipocytes. This speaks against an important role of ORP3 in the control of adipocyte differentiation. We previously characterized ORP3 as a phosphoprotein involved in cell adhesion, capable of modifying integrin activity, the actin cytoskeleton, and phagocytosis [41]. In contrast to the down-regulation of ORP3 we observed upon SGBS adipocyte differentiation, Lee et al. [58] found the protein up-regulated in adipocytes differentiated from human mesenchymal stem cells. The apparent inconsistency between their and our findings is most likely due to the use of different cell models: Consistent with the data in [58], ORP3 was also in our study among the ORPs expressed abundantly in the s.c. and visceral adipose depots, as well as in the SGBS adipocytes, even though we detected its moderate down-regulation during SGBS preadipocyte conversion into adipocytes. We thus find it likely that induction of ORP3 occurs in the adipocyte lineage between the mesenchymal stem cell and the preadipocyte stages [59]. Both ORP3 and ORP8 were down-regulated on the very first days of SGBS cell adipocytic differentiation. This may relate to the cessation of cell proliferation occuring at this stage – In other words, higher ORP3/ORP8 expression in the preadicytes could be associated with the active proliferation of these cells.

ORP2 was recently shown to have the capacity to facilitate cholesterol transfer from the plasma membrane to the endoplasmic reticulum and lipid droplets [43]. Moreover, we have earlier demonstrated localization of ORP2 on cytoplasmic lipid droplets [60]. However, in the present study we found the ORP2 mRNA down-regulated upon SGBS cell adipogenic differentiation, suggesting that elevated ORP2 expression is not essential for adipocyte lipid storage.

In summary, we report the expression patterns of OSBP/ORPs in human s.c. and visceral adipose depots and present, by using the SGBS preadipocyte/adipocyte model, the first data on the functional role of ORP proteins in adipocyte differentiation.

## Supporting Information

Figure S1 **Bioinformatic analysis of ORP mRNA expression in different tissues/cell types.** A heat map presentation based on the microarray data set of Human U133A/GNF1H Gene Atlas from BioGPS web site (http://biogps.org/downloads/ ).
(TIF)
