# Supplementary figures and images for: OSBP-Related Proteins (ORPs) in Human Adipose Depots and Cultured Adipocytes: Evidence for Impacts on the Adipocyte Phenotype
Source: PLoS One. 2012 Sep 21;7(9):e45352. doi: 10.1371/journal.pone.0045352 (PMC3448648; doi:10.1371/journal.pone.0045352)

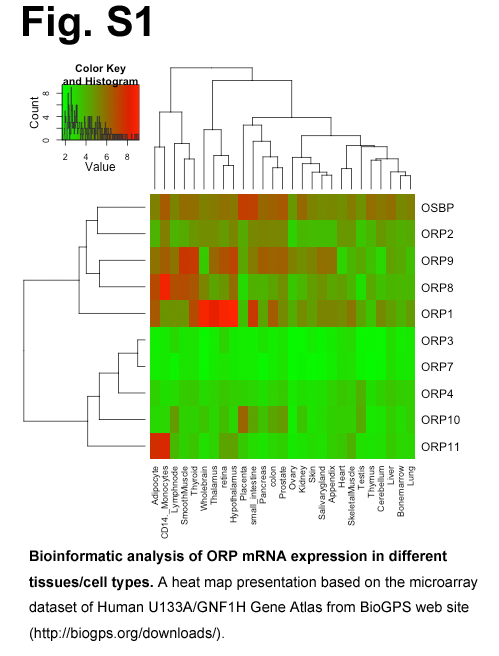

Supplement: Figure S1 — Bioinformatic analysis of ORP mRNA expression in different tissues/cell types. A heat map presentation based on the microarray data set of Human U133A/GNF1H Gene Atlas from BioGPS web site (http://biogps.org/downloads/ ). (TIF) [file pone.0045352.s001.tif]
